# Antidepressant-like activity of a brain penetrant HCN channel inhibitor in mice

**DOI:** 10.3389/fphar.2023.1159527

**Published:** 2023-05-10

**Authors:** Paulo Pinares-Garcia, James Spyrou, Chaseley E. McKenzie, Ian C. Forster, Ming S. Soh, Erlina Mohamed Syazwan, Mohammed Atif, Christopher A. Reid

**Affiliations:** ^1^ Florey Institute of Neuroscience and Mental Health, University of Melbourne, Parkville, VIC, Australia; ^2^ Epilepsy Research Centre, Department of Medicine, University of Melbourne, Heidelberg, VIC, Australia

**Keywords:** HCN channels, small molecule, antidepressant, depression, mood disorder, HCN channel block

## Abstract

Changes in Hyperpolarization-Activated Cyclic Nucleotide-Gated (HCN) channel function have been linked to depressive-like traits, making them potential drug targets. However, there is currently no peer-reviewed data supporting the use of a small molecule modulator of HCN channels in depression treatment. Org 34167, a benzisoxazole derivative, has been patented for the treatment of depression and progressed to Phase I trials. In the current study, we analysed the biophysical effects of Org 34167 on HCN channels in stably transfected human embryonic kidney 293 (HEK293) cells and mouse layer V neurons using patch-clamp electrophysiology, and we utilised three high-throughput screens for depressive-like behaviour to assess the activity of Org 34167 in mice. The impact of Org 34167 on locomotion and coordination were measured by performing rotarod and ledged beam tests. Org 34167 is a broad-spectrum inhibitor of HCN channels, slowing activation and causing a hyperpolarising shift in voltage-dependence of activation. It also reduced *I*_h_-mediated sag in mouse neurons. Org 34167 (0.5 mg/kg) reduced marble burying and increased the time spent mobile in the Porsolt swim and tail suspension tests in both male and female BALB/c mice, suggesting reduced depressive-like behaviour. Although no adverse effects were seen at 0.5 mg/kg, an increase in dose to 1 mg/kg resulted in visible tremors and impaired locomotion and coordination. These data support the premise that HCN channels are valid targets for anti-depressive drugs albeit with a narrow therapeutic index. Drugs with higher HCN subtype selectivity are needed to establish if a wider therapeutic window can be obtained.

## 1 Introduction

Depression is a leading cause of disability in the world (Herrman et al., 2022). Historically, treatment for major depressive disorder leads to remission in approximately one-third of patients and partial remission in another third (Souery et al., 2006; Warden et al., 2007) and, more recently, ketamine has been shown to be effective in treatment-resistant patients (Krystal et al., 2019; Samalin et al., 2022). However, more than 50% of patients that are trialled on ketamine do not go into remission, with many suffering adverse events including sedation, somnolence, anxiety and cardiovascular episodes (Short et al., 2018; Samalin et al., 2022). Therefore, there remains an unmet need for efficacious, on-target therapeutic strategies for patients with treatment-resistant depression.

Hyperpolarization-activated cyclic nucleotide-gated (HCN) channels are members of the voltage gated channel superfamily (Yu and Catterall, 2004; Wahl-Schott and Biel, 2009; Benarroch, 2013). There are four isoforms encoded by *HCN1, HCN2, HCN3* and *HCN4*. Each isoform has distinct expression profiles, kinetics, activation voltages, and cyclic AMP sensitivity (Biel et al., 2009). HCN channels are activated by hyperpolarisation and carry a non-selective cation conductance giving rise to *I*_h_ (Biel et al., 2009). These unique properties allow them to control a range of neuronal functions, including setting the resting membrane potential, modulating the integration of dendritic synaptic input, reducing neuronal input resistance, and regulating pacemaker activity (Surges et al., 2004; Biel et al., 2009). HCN channels have been implicated in a range of neurological conditions and are proposed as potential molecular targets in areas such as neuropathic pain, cognitive impairment and affective disorders (Postea and Biel, 2011; DiFrancesco and DiFrancesco, 2015; Ramirez et al., 2018; Santoro and Shah, 2020).

Of the four isoforms, HCN1 channels have been specifically implicated in depression (Shah, 2012; Ku and Han, 2017; Kim and Johnston, 2018). *Hcn1* knockout mice spend more time mobile in the Porsolt swim and tail suspension tests that are used routinely as preclinical models to test for antidepressant activity (Lewis et al., 2011). Similarly, knockout of *Trip8b*, an auxiliary protein, reduces both HCN1 and HCN2 channel expression, and causes mice to spend more time mobile in the Porsolt swim and tail suspension tests (Lewis et al., 2011). More targeted approaches including the viral delivery of shRNA to knockdown HCN1 in the CA1 hippocampal region also enhanced mobility in the Porsolt swim test (Kim et al., 2012). Furthermore, HCN1 channel expression is increased in the CA1 region of the dorsal hippocampus in a rat model of chronic unpredictable stress. Importantly, the stress response was decreased when HCN1 was knocked down using an shRNA approach (Kim et al., 2018). The case for HCN2 is less straightforward. *Hcn2* knockout mice spend more time mobile in the tail suspension test (Lewis et al., 2011). However, expression of HCN2 is reduced in cholinergic interneurons in the nucleus accumbens of mice subjected to chronic stress, with overexpression sufficient to rescue the depressive phenotypes (Cheng et al., 2019). Similarly, HCN2 overexpression in dopamine neurons of the ventral tegmental area was effective in reversing the depressive phenotypes caused by chronic mild unpredictable stress in mice (Zhong et al., 2018). While the picture is not clear for HCN2 channels, these data strongly support the idea that HCN1 channel inhibition may be an effective therapeutic approach to treat depression. Here we investigate the effects of Org 34,167, a broad-spectrum brain penetrant HCN channel inhibitor, on mouse screens of depressive-like behaviour.

Org 34,167 is a propyl-1,2 benzisoxazole derivative, initially synthesised by Organon Laboratories, that has been patented as a drug for the treatment of depression (Patent WO 97/40,027) (Leysen et al., 1997). Our knowledge of the physiological impact of Org 34,167 is limited to a single report of its efficacy in a rat model useful for the study of absence epilepsy (Samotaeva et al., 2013) and its characterisation in a published PhD thesis by D.A. Slattery (Slattery, 2003). Slattery reports that Org 34,167 is potent in a number of animal models useful for the study of depression, and also states that Org 34,167 completed phase 1 trials and was well tolerated at low doses (<2.5 mg), with only moderate side effects seen at doses as high as 40 mg (Slattery, 2003). Here, we show that Org 34,167 is effective at reducing depressive behaviours in three mouse behavioural assays. The drug was well tolerated at effective anti-depressive doses, although tremors were noted at higher doses. These data support the idea that small molecules that inhibit HCN channels may be effective antidepressants.

## 2 Materials and methods

### 2.1 Cell culture

HEK293 cells stably transfected with human HCN1 (hHCN1; Catalog #CT6114) and hHCN4 (Catalog #CT6006; Charles River Laboratories, Cleveland, OH, United States) were maintained in high glucose DMEM (Gibco, Grand Island, NY, United States), supplemented with 10% v/v Tet-System Approved Fetal Bovine Serum (FBS; Gibco), 1% penicillin/streptomycin (P/S) solution, 5 μg/mL Blasticidin and 100 μg/mL Zeocin. HEK293 cells stably transfected with hHCN2 (Catalog #CYL3041; Eurofins, St Charles, MO, United States) were maintained in high glucose DMEM (Gibco), 10% FBS (Invitrogen, Carlsbad, CA, United States), supplemented with 1% P/S solution, and 400 μg/mL Geneticin (G418 Sulfate; Gibco). All cells were maintained at 37°C and 5% CO_2_ and grown to ∼80% confluency in T25 flasks (BD Biosciences, San Jose, CA, USA) before being passaged or harvested for electrophysiological recordings. Expression of hHCN1 and hHCN4 were induced by the addition of 1.5 μg/mL tetracycline (Sigma-Aldrich, Castle Hill, NSW, Australia) to cells at least 24 h prior to patch-clamp recording.

### 2.2 Patch-clamp electrophysiology

HEK293 cells were detached from flasks with 0.05% Trypsin/EDTA (Gibco) and resuspended in extracellular buffer solution. The extracellular solution contained (in mM) 110 NaCl, 30 KCl, 2 CaCl_2_, 1 MgCl_2_, 10 HEPES and 5 D-Glucose, at pH 7.4 (adjusted with NaOH) and 298 mOsm. The intracellular solution contained (in mM) 10 NaCl, 50 KCl, 60 KF, 1 CaCl_2_, 1 MgCl_2_, 10 HEPES and 20 EGTA, at pH 7.2 (adjusted with KOH) and 285 mOsm. High quality seals were obtained by the temporary addition of a seal enhancer solution, which contained (in mM) 80 NaCl, 3 KCl, 35 CaCl_2_, 10 MgCl_2_ and 10 HEPES, at pH 7.4 (adjusted with HCl) and 298 mOsm. All three buffer solutions were made with Type 1 Ultrapure MilliQ H_2_O (Merck Millipore, Darmstadt, Germany) and filtered with a 0.22 µm vacuum filter (Corning, Acton, MA, United States).

Suspended cells were placed in the cell hotel of a Patchliner (Nanion Technologies, Munich, Germany) and cooled to 15 °C with a Patchliner Cooling Plate. Regular automatic pipetting was used to maintain cells in suspension. Planar NPC-16 chips (Nanion Technologies) with one hole/well and a resistance of 1.8–3 MΩ/hole were used, and recordings were acquired with PatchMaster version 2x90.4 (HEKA Instruments Inc., Bellmore, NY, United States). Recordings were processed and analysed with MATLAB R2020a (MathWorks, Natick, MA, United States) and GraphPad Prism 9 (GraphPad, San Diego, CA, United States).

The voltage-dependent activation of HCN channels was assessed using a step protocol by which cells were hyperpolarised to a range of potentials from −40 mV to −130 mV (or −140 mV for cells expressing hHCN4) in −10 mV increments, from a holding potential of −30 mV, for a total of 10 sweeps for cells expressing hHCN1 or hHCN2 or 11 sweeps for cells transfected with hHCN4, with a wait time of 2 s between sweeps. Org 34,167 (SYNthesis Med Chem, Shanghai, China) was dissolved in 0.1% dimethyl sulfoxide (DMSO; Sigma-Aldrich) in external buffer solution. Two sets of recordings were made for each cell; the first with cells exposed to external solution only, and the second with cells exposed to varying concentrations of Org 34,167. Leak subtraction of tail currents was performed by subtracting the value of the current at −40 mV from all current values. Instantaneous tail currents for each individual sweep were measured and normalised to values between 0 and 1, where 0 is set as the tail current of the first sweep (at −40 mV) and 1 is set as the tail current for the final sweep (−130 mV for HCN1 and HCN2; −140 mV for HCN4). The normalised tail current values at −110 mV, both before and after the addition of Org 34,167, were used to calculate proportion inhibition by Org 34,167 as (*I*_*Buffer*_–*I*_*Org*_)/*I*_*Buffer*_ for each HCN channel at each concentration of Org 34,167. These proportional inhibition values were then used to produce and analyse dose response curves by non-linear regression for the effect of Org 34,167 on each HCN channel isoform, with the maximum for each curve set to 100%, the minimum set to 0%, and the Hill slope value set to −1. (GraphPad Prism 9, sigmoidal dose response, variable slope, least squares fit).

The voltage of half-maximal activation (*V*_0.5_) were calculated by fitting the normalised current values at each sweep with the following modified Boltzmann equation:

$$I\left(V\right)=1/\left(1+e^{\frac{V-V_{0.5}}{k}}\right)$$

Where *V* is the voltage, *I*(*V*) is the current at that voltage, *V*_0.5_ is the half-maximal activation point, and *k* is the slope factor. Δ*V*_0.5_ values for each cell were calculated by subtracting the buffer *V*_0.5_ from the *V*_0.5_ following treatment with Org 34,167, which were then used to produce dose response curves by non-linear regression with the minimum for each curve set to 0 (GraphPad Prism 9, sigmoidal dose response, variable slope, least squares fit). Voltage-dependence of activation time constants (Tau^act^) were obtained from fitting activation current traces a with single-exponential function, starting after the initial inflection.

### 2.3 Animal housing, husbandry and welfare

Animals were housed in standard 15 × 30 × 12 cm cages, maintained under 12-h dark and light cycles in a temperature-controlled room, and had access to dry pellet food and tap water *ad libitum*. Post-weaning P21 male C57BL/6J (IMSR Cat# JAX_000664, RRID: IMSR_JAX:000,664) or male and female BALB/c (IMSR Cat# JAX_000651, RRID: IMSR_JAX:000,651) mice were ordered from the Animal Resources Centre (ARC; WA, Australia). Mice were monitored daily and were killed by rapid cervical dislocation, an ANZCCART-approved method (Australian and New Zealand Council for the Care of Animals in Research and Teaching, 2001).

### 2.4 Brain slice electrophysiology

Brain slice electrophysiological recordings were performed as previously described (Bleakley et al., 2021). Female BALB/c mice (P49-56) were deeply anaesthetised using isoflurane. Mice were decapitated, and their brains were immediately removed and mounted in a slice chamber with ice-cold cutting solution, bubbled continuously with carbogen gas. Cutting solution contained (in mM) 125 choline chloride, 20 D-Glucose, 0.4 CaCl_2_·2H_2_O, 6 MgCl_2_·6H_2_O, 2.5 KCl, 1.25 NaH_2_PO_4_ and 26 NaHCO_3_.

300 µm thick coronal slices were cut on a vibratome (Leica VT1200S), which were then transferred to and maintained in a holding chamber filled with artificial cerebrospinal fluid (aCSF), which contained (in mM) 125 NaCl, 10 D-Glucose, 2 CaCl_2_·2H_2_O, 2 MgCl_2_·6H_2_O, 2.5 KCl, 1.25 NaH_2_PO_4_ and 26 NaHCO_3_. The electrode internal solution for all recordings contained (in mM) 125 K-gluconate, 5 KCl, 2 MgCl_2_·6H_2_O, 10 HEPES, 4 ATP-Mg, 0.3 GTP-Na, 10 phosphocreatine, 0.1 EGTA and 0.2% biocytin; pH 7.2. aCSF was bubbled continuously with carbogen gas for 30 min at 32°C and for at least an additional 30 min at room temperature prior to recording.

Brain slices were mounted in a recording chamber and continually perfused with aCSF, with a temperature of 32°C and bubbled with carbogen gas (2 mL/min). Borosilicate glass electrodes with initial resistances of 3–7 MΩ were used to perform whole-cell patch clamp recordings from layer V pyramidal neurons in medial prefrontal cortex (mPFC). These recordings were made with Axon Multiclamp 700B amplifier (Molecular Devices), Digidata 1550 digitizer (Molecular Devices), and pCLAMP version 10 software. All slice electrophysiology data were sampled at 50 kHz with a low-pass filter at 10 kHz, collected in current clamp, and analysed using AxoGraph X (version 1.7.6).

Sag was elicited in layer V neurons with a −70 pA current injection, from a −70 mV holding potential, in the presence and absence of Org 34,167 (30 mM), which was washed on for at least 5 min. Sag was defined as the difference between the most hyperpolarised point within the first second and the final “steady state” of the voltage trace.

### 2.5 Pharmacokinetic studies

Formulations were prepared at concentrations of 0.05 and 0.5 mg/mL by dissolving solid Org 34,167 in 0.9% (w/v) saline solution. Mice were injected IP (dose volume 10 mL/kg) and blood samples were collected up to 24 h (*n* = 3 mice per time point) with a maximum of three samples from each via submandibular bleed (approximately 120 μL; conscious sampling).

Blood was collected into Eppendorf tubes containing heparin and stabilisation cocktail (containing Complete^®^ (a protease inhibitor cocktail) and potassium fluoride). Blood samples were centrifuged immediately, supernatant plasma was removed, and stored at −80°C until analysis by liquid chromatography-mass spectrometry (LC-MS). Whole brains were blotted to remove excess blood, placed into pre-weighed polypropylene vials, and weighed. The brains were snap frozen in dry ice and subsequently stored frozen (−80°C) until analysis.

Plasma samples were quantified against calibration standards prepared by spiking blank mouse plasma (50 µL) with solution standards (10 µL) obtained by diluting a stock solution of test compound (1 mg/mL in DMSO) with 50% (v/v) acetonitrile in water. Diazepam (10 µL of 5 μg/mL in 50% acetonitrile/water) was added to all plasma calibration standards and samples as an internal standard (IS). Protein precipitation was carried out by the addition of acetonitrile followed by vortexing and centrifugation (10,000 rpm) for 3 min to obtain supernatant for analysis using the LC-MS conditions.

Pre-weighed brain tissue samples were homogenised using a glass rod in buffer containing an EDTA/potassium fluoride stabilisation cocktail (3 mL cocktail/g tissue). An aliquot (200 µL) was transferred to a fresh Eppendorf tube for sample extraction. Tissue homogenate standards were prepared by spiking blank homogenate (200 µL containing 50 mg tissue) with solution standards (10 µL) and the internal standard, diazepam (10 μL, 5 μg/mL). The test samples were similarly prepared, except that blank acetonitrile (10 µL) was added instead of solution standards. Protein precipitation was carried out by the addition of acetonitrile (600 µL), vortexing for 20 s, and centrifugation (10,000 rpm) in a microcentrifuge for 3 min. The supernatant was subsequently separated for LC-MS.

### 2.6 Behavioural tests

Prior to all behavioural experiments, mice were acclimatized for 1 h in a dimly lit behavioural room. Mice underwent each behavioural test 30 min after an intraperitoneal (IP) injection with Org 34,167 or vehicle (0.9% saline). Similarly, as a positive control, mice underwent the marble burying, Porsolt swim test or tail suspension test 30 min after an IP injection with fluoxetine or vehicle.

#### 2.6.1 Open field exploratory locomotion assay

Mice were placed in a square 27.3 cm × 27.3 cm × 20.3 cm open-field arena and were allowed to move freely for 30 min, during which infrared emitters and detectors tracked their movement. Data indicating distance travelled, time active and other parameters were recorded and compiled using MED Associates Activity Monitor software (Med Associates Inc.)

#### 2.6.2 Rotarod

Mice were trained to walk on the rotarod over three sessions: two 2-min sessions at a fixed speed of 4 rpm, and one 2-min session accelerating from 4 to 20 rpm. Mice were then tested on the rotarod for three 10-min trials (with at least 30 min between trials). Each trial consisted of 5 min where the rotarod accelerated from 4 to 40 rpm, followed by 5 min at a fixed speed of 40 rpm. Latency to fall was recorded. Total time on the rotarod (maximum 1,800 s) was summed and analysed.

#### 2.6.3 Ledged beam assay

Mice were trained over two trials to traverse a ledged beam placed at a 15-degree angle 90 cm above the ground. The ledged beam consisted of a 1-m-long plastic beam which tapers from a width of 33–1 mm, with underhanging ledges that were 5 mm wide and positioned 10 mm below the upper surface of the beam. Mice completed four test trials where they were videoed as they traversed the final 50 cm of the beam. Average “foot faults” (where mice stepped on the underhanging ledge, scored manually) and average time taken to complete the test were calculated for each mouse, excluding trials where the mouse turned around and descended the beam.

#### 2.6.4 Porsolt swim test

Mice were placed individually in transparent cylindrical glass cylinders (25 cm high and 15 cm in diameter) with a water depth and temperature of 15 cm and 23°C–25°C, respectively. The activity of the mice was observed during a period of 6 min using a small animal behaviour analysis system (MedAssociates), and the duration of immobility over the last 4 min of the 6-min period was manually scored by two experienced observers blinded to treatment groups. Mice were judged to be immobile when they stopped struggling and floated on the water or made only small movements necessary to keep its head above water.

#### 2.6.5 Tail suspension test

The end of the tail of each mouse (2 cm from the tip) was fixed with medical tape to a hook attached to the suspension apparatus so that the mouse was hanging upside down with its head approximately 5 cm off the ground. The activity of the animals was recorded over 6 min, and the duration of immobility over the last 5 min of the 6-min period was manually scored by two experienced observers blinded to the treatment groups. Mice were considered immobile only when completely motionless and mice that climbed their tails were excluded from the data.

#### 2.6.6 Marble burying test

Transparent glass cages (30 cm × 18 cm × 19 cm) were filled with an approximately 5-cm-deep layer of sawdust. 20 glass marbles per cage (1.5 cm diameter) were then evenly spaced in a perimeter 2 cm away from the wall of the cage. During the testing phase, each mouse was placed in the cage and allowed to explore it for 20 min. At the end of the test, mice were removed from the cage and the number of marbles buried with bedding up to at least two-thirds of their depth was manually counted and independently scored by two blinded investigators. The entire test session was also recorded on video for later analysis.

#### 2.6.7 Heart rate analysis

Heart rate measurements were collected using MouseOx^®^ recording collars and MouseOx^®^ Plus Software (Version 1.6; STARR Life Sciences, Alleghany, PA, United States) in 17 cm × 14 cm clear plastic chambers. BALB/c mice were acclimatised to the chambers and collars for 15 min, after which baseline heart rate recordings were taken for 15 min. Mice then had their collars removed, were removed from their chambers, and were injected IP with either Org 34,167 (0.5 or 1.0 mg/kg; *n* = 7 each) or vehicle (0.9% saline; *n* = 6). Mice were then re-acclimatised to their collars and chambers for 10 min, after which post-treatment heart rate recordings were taken for 35 min. During the entire testing duration, an experimenter was in the room with the animals. These measurements were used to calculate average heart rate for each treatment group over the final 35-min period.

### 2.7 Statistics

Results were considered statistically significant at *p*-values <0.05. ANOVAs and Dunnett’s post-hoc tests were performed using GraphPad Prism 9. ANOVA statistical summaries and reporting can be found in Supplementary Table S2. All data are presented as mean ± SEM unless stated otherwise.

### 2.8 Study approval

All experiments were performed in accordance with the Prevention of Cruelty to Animals Act, 1986, under the guidelines of the National Health and Medical Research Council (NHMRC) Code of Practice for the Care and Use of Animals for Experimental Purposes in Australia and were approved by the Animal Ethics Committee at the Florey Institute of Neuroscience and Mental Health. PK studies performed at MIPS (Monash Institute of Pharmaceutical Sciences) were in accordance with the relevant guidelines and standard operating procedures (SOPs) of the Centre for Drug Candidate Optimisation, Monash University. Animal studies are reported in compliance with the ARRIVE guidelines (Kilkenny et al., 2010).

## 3 Results

**FIGURE 1:**
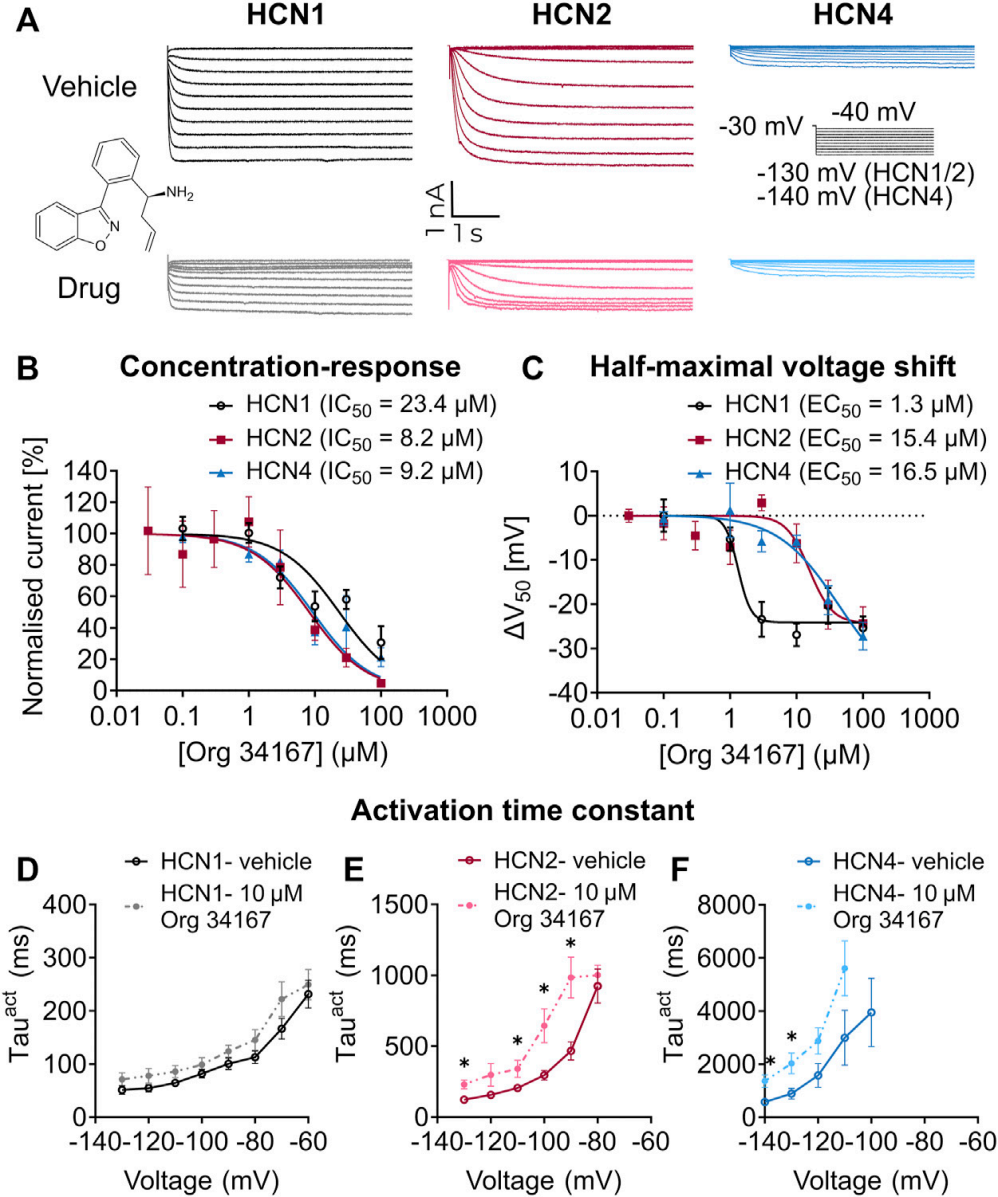
Effect of Org 34,167 on HCN channels in HEK293 cells. **(A)** Representative steady-state current traces for vehicle (top panel) and 10 µM Org 34,167 (bottom panel) on HCN1, HCN2 and HCN4 channels, elicited from the activation protocol shown in between the HCN4 traces. Chemical structure of Org 34,167 inset between panels. **(B)** Org 34,167 dose-response curves (0.03–100 µM) and IC_50_ values in HEK293 cells expressing HCN1, HCN2 or HCN4 channels. The current *(I)* data of independent measurements consists of proportional inhibition values of Org 34,167 calculated from tail current measurements at −110 mV, normalised to untreated controls. **(C)** Org 34,167 dose-response curves of Δ*V*_0.5_ (shifts in the voltage of half-maximal activation) in HEK293 cells expressing human HCN1, HCN2 or HCN4 channels. The Δ*V*_0.5_ values were calculated from normalised current data before and after Org 34,167 administration for each individual cell. **(D–F)** Voltage-dependence of activation time constants (Tau^act^) of **(D)** HCN1, **(E)** HCN2 or **(F)** HCN4 channels in the absence or presence of 10 µM Org 34,167, obtained from fitting activation current traces with single-exponential function. *N* = 2–19 cells per concentration per channel for **(A–F)**. Data represented as mean ± S.E.M, with individual values where relevant. *p < 0.05.

**FIGURE 2:**
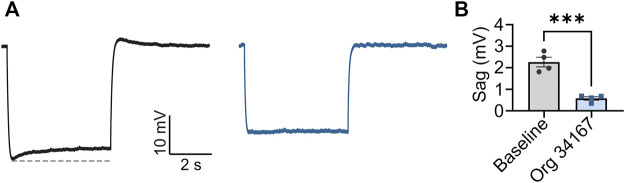
Org 34,167 (30 µM) significantly reduces sag in layer V pyramidal neurons. **(A)** Average of sag traces obtained from cells from female BALB/c mice (*n* = 4) before (black) and after (blue) Org 34,167 administration from slice electrophysiology recordings. Dotted line (grey) indicates maximal sag. Sag was elicited with **(A)** 70 pA current injection from **(A)** 70 mV holding membrane potential (Vm). **(B)** Comparison of sag before and after Org 34,167 administration (*n* = 4). ***p < 0.001.

Org 34,167 is a propyl-1,2 benzisoxazole derivative that is reported to be a blocker of HCN channels (Slattery, 2003; Samotaeva et al., 2013) (Figure 1A). To confirm this, we applied Org 34,167 externally to HEK293 cells expressing recombinant human HCN1, HCN2 or HCN4 channels. Voltage-current relationships were determined using whole-cell voltage clamp recordings with robust currents observed for all HCN channel subtypes (Figure 1A, Supplementary Figure S1). Dose-response curves were constructed by measuring the normalised inhibition at −110 mV, a potential where currents were most stable, and activation was maximal. Org 34,167 inhibited all HCN channel subtypes in a dose-dependent manner (Figures 1A, B). The potency of Org 34,167 against HCN2 and HCN4 channels was similar, with HCN1 channels having a higher IC_50_ for the drug at currents recorded at −110 mV (Figure 1B). Org 34,167 caused a significant dose-dependent hyperpolarising shift in the voltage of half-maximal activation (*V*_0.5_; Figure 1C). Interestingly, the potency of Org 34,167 was greater for HCN1 channels for this parameter (1.3 µM), whereas HCN2 and HCN4 were similar (15.4 and 16.5 µM, respectively). Application of 10 µM Org 34,167 also slowed the activation of all HCN channels (Figure 1D–F). Furthermore, brain slice electrophysiology recordings revealed that Org 34,167 (30 µM) significantly reduced *I*_h_-mediated sag in layer V mPFC pyramidal neurons in female BALB/c mice (Figure 2). These data highlight robust inhibition of human and mouse HCN1, HCN2 and HCN4 channels by Org 34,167 through a variety of biophysical mechanisms.

To determine drug potency in a whole animal model, we next investigated the pharmacokinetic profile of Org 34,167 in C57BL/6J mice. Mice were injected intraperitoneally (IP) at 0.5 and 5 mg/kg and blood samples were taken at regular intervals and plasma levels measured. At 5 mg/kg Org 34,167, all mice started to display tremors and sampling at this dose was terminated. At a dose of 0.5 mg/kg, the plasma levels of Org 34,167 peaked within 15 min and decayed with a half-life of 46.8 min (Supplementary Figure S2). Additional pharmacokinetic parameters are presented in Supplementary Table S1. In a subset of mice given a dose of 5 mg/kg, brains were removed 37 min post-injection; Org 34,167 levels were 16.9 ± 5.8 (*n* = 3) times greater in the brain (10.57 ± 3.43 µM) than in the plasma (0.649 ± 0.051 µM), which confirmed its brain penetrance.

**FIGURE 3:**
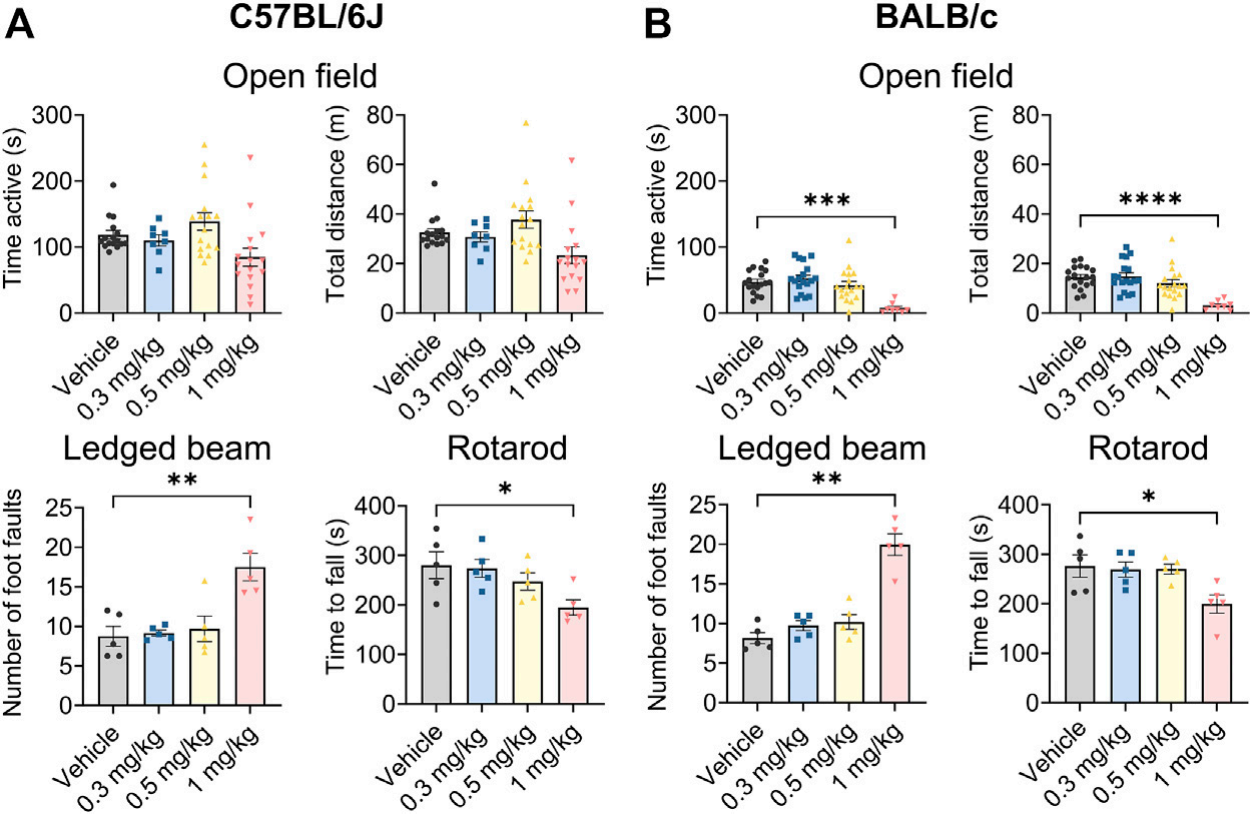
Behavioural characterisation of Org 34,167 (0.3, 0.5, and 1 mg/kg) in male C57BL/6J and BALB/c mice. **(A)** Effect of Org 34,167 on time active and total distance travelled in open field test (*n* = 8–16/group), number of foot faults in ledged beam test (*n* = 5/group), and time to fall in rotarod test (*n* = 5/group), in male C57BL/6J mice. **(B)** Effect of Org 34,167 on male BALB/c mice on time active and total distance travelled in open field test (*n* = 8–18/group), number of foot faults in ledged beam test (*n* = 5/group), and time to fall in rotarod test (*n* = 5/group). Data represented as individual values and mean ± S.E.M. *p < 0.05, **p < 0.01, ***p < 0.001, ****p < 0.00001, one-way ANOVA with Dunnett’s post-hoc.

Org 34,167 was tested at a range of doses in standard behavioural assays used to broadly assess toxicology in both C57BL/6J and BALB/c mice. Org 34,167 was well tolerated in C57BL/6J mice at doses up to 0.5 mg/kg in the open field, ledged beam and rotarod tests (Figure 3A). At 1 mg/kg the number of foot faults increased, and mice fell off the rotarod more quickly relative to vehicle control, indicative of drug toxicity. Similarly, in BALB/c mice Org 34,167 was well tolerated up to 0.5 mg/kg (Figure 3B). At 1 mg/kg male BALB/c mice were less active and covered less distance in the locomotor test, displayed more foot faults in the ledged beam test and fell earlier on the rotarod test relative to vehicle control mice (Figure 3B). Interestingly, for female BALB/c mice, only performance in the rotarod test was observed to be significantly impacted (Supplementary Figure S3). As noted above, tremors were observed at 5 mg/kg in the pharmacokinetic studies. Visually scored tremors were also noted in a small proportion of mice at 1 mg/kg (approximately 22% of C57BL/6J mice and 28% of BALB/c). These data suggest that Org 34,167 was less well-tolerated at this dose and in light of this, all subsequent behavioural testing was done at doses at or below 0.5 mg/kg.

**FIGURE 4:**
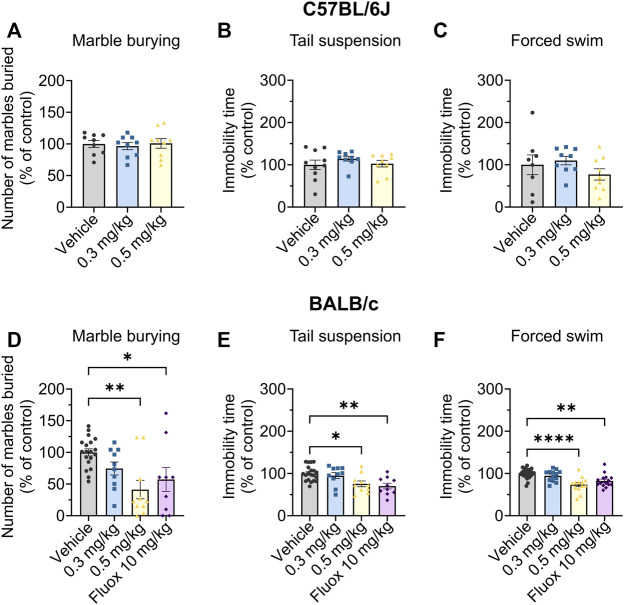
Marble burying, tail suspension and Porsolt swim test data in male C57BL/6J (top) and BALB/c (bottom) mice. Effect of Org 34,167 (0.3 and 0.5 mg/kg) and/or fluoxetine (10 mg/kg; Fluox) as positive control on number of marbles buried by male **(A)** C57BL/6J and **(D)** BALB/c mice, and on the immobility time in **(B, E)** tail suspension and **(C, F)** Porsolt swim test in male **(B, C)** C57BL/6J and **(E, F)** BALB/c mice. Data are represented as individual values and mean ± S.E.M (n ≥ 10/group; *p < 0.05, **p < 0.01, ****p < 0.0001; one-way ANOVA with Dunnett’s post-hoc).

The marble burying assay is routinely used as an acute screening tool for testing potential anxiolytic and antidepressant compounds (Nicolas et al., 2006), with a reduction in marbles buried being an indicator of reduced anxiety and/or depression. To allow comparisons across multiple cohorts in this assay we normalised the drug response within each cohort, setting the average vehicle control to 100%. Org 34,167 did not change the number of marbles buried by C57BL/6J mice at doses up to 0.5 mg/kg (Figure 4A). However, Org 34,167 (0.5 mg/kg) was effective at reducing marble burying in male BALB/c mice, indicating antidepressant/anxiolytic activity, and had a similar efficacy to fluoxetine (10 mg/kg; Figure 4D), a selective serotonin reuptake inhibitor (SSRI) used clinically for depression (Wong et al., 1995; Rossi et al., 2004).

As such, BALB/c mice were the focus of subsequent behavioural testing. This is in line with previous studies which illustrate that BALB/c mice show adequate antidepressive-like effects in response to fluoxetine, whereas other strains, including C57BL/6J, are insensitive to SSRIs or display ceiling effects in response to them. Further, BALB/c mice display consistent responses to behavioural tests of despair and are believed to better reflect and recapitulate the features of clinical depression seen in human patients (Lucki et al., 2001; Jacobson and Cryan, 2007; McVey Neufeld et al., 2018).

Next, we used two well established antidepressant preclinical screens, the Porsolt swim and tail suspension tests. In these tests, increased mobility is indicative of a reduced susceptibility to despair which correlates with depressive traits (Porsolt et al., 1977; Steru et al., 1985). In both tests, Org 34,167 (0.5 mg/kg) increased mobility consistent with antidepressant-like activity in male BALB/c mice (Figures 4E, F). The efficacy of Org 34,167 (0.5 mg/kg) was similar to that observed with fluoxetine in both cases.

**FIGURE 5:**
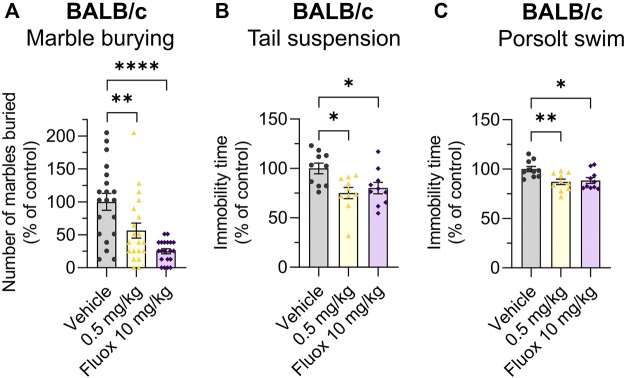
Marble burying, tail suspension and Porsolt swim test data in female mice. Effect of Org 34,167 (0.5 mg/kg) and/or fluoxetine (10 mg/kg; Fluox) as positive control on **(A)** number of marbles buried by female BALB/c mice, and on the immobility time in **(B)** tail suspension and **(C)** Porsolt swim test in female BALB/c mice. Data represented as individual values and mean ± S.E.M (n ≥ 10/group; *p < 0.05, **p < 0.01, ****p < 0.0001; one-way ANOVA with Dunnett’s post-hoc).

The effects of Org 34,167 on the marble burying, Porsolt swim and tail suspension tests were repeated in female BALB/c mice. As observed in male mice, a dose of 0.5 mg/kg significantly reduced the number of marbles buried and increased the time spent mobile (Figures 5A–C). Collectively, the efficacy of Org 34,167 in three acute affective disorder tests strongly suggests that inhibition of HCN channels can reduce depressive-like behaviour in male and female mice. Consistent with what was observed in the marble burying test, in C57BL/6J mice Org 34,167 did not reduce immobility time (Figures 4B, C).

**FIGURE 6:**
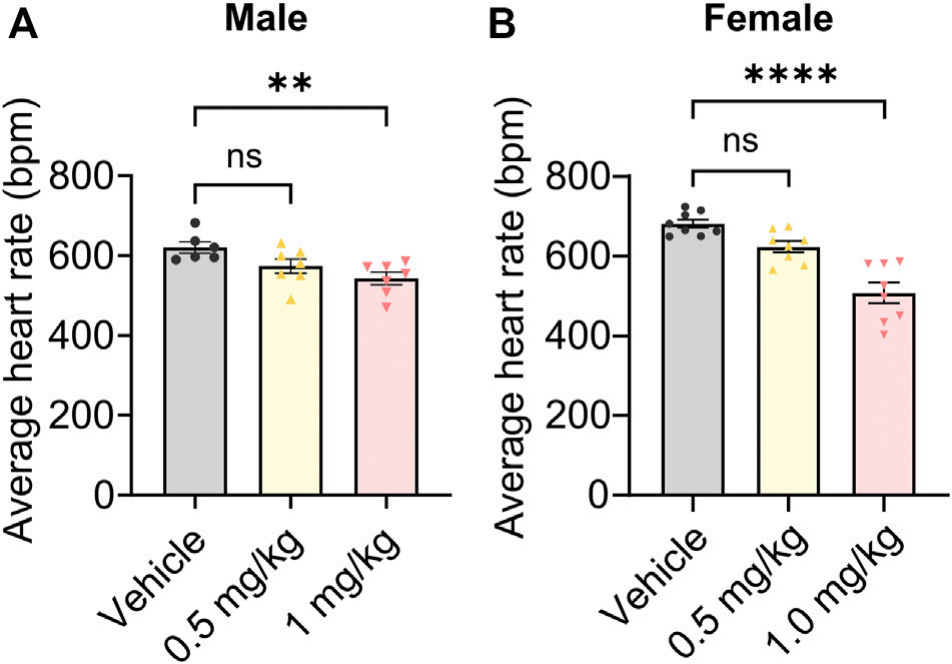
Impact of Org 34,167 on heart rate. Effect of Org 34,167 (0.5 and 1 mg/kg) on the average heart rate of male **(A)** and female **(B)** BALB/c mice over a 35-min monitoring period. Data represented as individual values and mean ± S.E.M (*n* = 6–8/group; **p ≤ 0.01, ****p ≤ 0.0001, ns, not significant; one-way ANOVA with Dunnett’s post-hoc).

Finally, we probed potential on-target toxicity in mice. HCN channels are highly expressed in the heart, where they are responsible for regulating heart rate (Hennis et al., 2022). Org 34,167 significantly reduced heart rate only at a dose of 1.0 mg/kg, with heart rate being unaffected at 0.5 mg/kg in both male (Figure 6A) and female (Figure 6B) mice.

## 4 Discussion

HCN channels have been strongly implicated in the underlying pathology of depression (Lewis et al., 2011; Kim et al., 2012; Han et al., 2017; Lyman et al., 2017; Kim et al., 2018; Kim and Johnston, 2018; Cheng et al., 2019; Luo et al., 2019; Santoro and Shah, 2020). However, reports of the utility of a small molecule inhibitor of HCN channels in depression are to the best of our knowledge limited to one thesis (Slattery, 2003). Org 34,167 is patented for use in depression and is reported to be well tolerated in humans (Patent Application WO 97/40,027) (Leysen et al., 1997; Slattery, 2003). Here we confirm that Org 34,167 is a robust inhibitor of the predominant HCN channel isoforms expressed in the brain (Oyrer et al., 2019). The pharmacokinetic profile in mice is favourable and conducive to performing behavioural experiments. Furthermore, the drug concentrates in the brain at a level ∼17 times greater than that observed in plasma. Org 34,167 (0.5 mg/kg) reduced depressive-like behaviours in male and female mice in three separate preclinical tests. The improvement in depressive-like behaviours was similar to that noted for fluoxetine at 10 mg/kg. At 0.5 mg/kg, Org 34,167 did not cause any overt toxicity, but a slight increase in dose to 1 mg/kg resulted in a detrimental impact on mouse behaviour across the open field, ledged beam and rotarod tests. Furthermore, tremors emerge in ∼25% of mice at the 1 mg/kg dose. These data suggest that Org 34,167 has utility as an antidepressant drug, but with a narrow therapeutic window. Our results will motivate additional studies investigating more subtype-selective compounds in depression to assess if it is possible to widen this therapeutic window.

Org 34,167 is a broad-spectrum inhibitor of HCN channels with a complex impact on the biophysical properties of these channels. This includes apparent inhibition, a hyperpolarising shift in the voltage dependence of activation, and a slowing of the activation of the channel, thereby diminishing the contribution of HCN channels to membrane conductance. Org 34,167 was marginally more selective for HCN2 and HCN4 over HCN1 channels based on the percentage inhibition at −110 mV. Interestingly, the hyperpolarising shift in the voltage of activation was very sensitive to Org 34,167, with an EC_50_ almost 20 times lower than the percentage inhibition of the HCN1 channel. Our pharmacokinetic studies predict a brain concentration of ∼1.7 μM at a 0.5 mg/kg dose in mice. This suggests that, at the doses used to achieve anti-depressive effects in mice, the major biophysical impact would be a shift in the voltage of activation of HCN1 channels that has an EC_50_ of 1.3 µM for this parameter.

Physiological experiments also point to HCN1 channel activity as an important driver of pathology in depression. This includes increased mobility in the Porsolt swim and tail suspension tests for the *Hcn1* knockout mouse and in mice where dorsal hippocampal HCN1 channels are specifically knocked down (Lewis et al., 2011; Kim et al., 2012). Furthermore, chronic stress increased hippocampal HCN1 channel expression with a knock-down strategy sufficient to reverse any deficits (Kim et al., 2018). Interestingly, an increase in *I*_*h*_ in ventral tegmental area (VTA) dopaminergic neurons has also been observed in a mouse model of chronic social defeat stress (Cao et al., 2010). A local infusion of ZD7288 or DK-AH 269 (cilobradine), broad-spectrum HCN channel inhibitors used as bradycardic agents, into the VTA of these mice has been shown to reverse stress-induced social avoidance with a similar efficacy to chronic fluoxetine administration (Cao et al., 2010). *Hcn2* knockout mice have anti-depressive behaviours (Lewis et al., 2011). However, knockdown of HCN2 channels in cholinergic and dopaminergic neurons are associated with chronic stress, with depressive-like behaviours in these mice reversed by the selective upregulation of the channel (Zhong et al., 2018; Cheng et al., 2019). Whilst hippocampal HCN4 channel knockdown has been proposed to promote anxiety-like activity (Gunther et al., 2019), there was a subtle anxiolytic effect seen in brain-specific *Hcn4* knockout mice (Kharouf et al., 2020). The impact of HCN4 channels on depressive behaviour is currently unknown. Collectively, these findings suggest that compounds more selective for HCN1 channels will plausibly have a greater anti-depressive efficacy.

The two primary side effects noted in mice are tremors and reduced heart rate. These are both likely to be due to on-target effects. *Hcn2* knockout mice are reported to have severe tremors (Ludwig et al., 2003), whereas *Hcn1* and *Hcn4* knockout mice are tremor-free (Nolan et al., 2003; Zobeiri et al., 2019; Kharouf et al., 2020). Therefore, compounds that have less activity against HCN2 channels are expected to have a lower propensity to cause tremors. A reduced heart rate is to be expected, given that other broad-spectrum HCN channel modulators, including ivabradine, are known to cause a self-limiting bradycardia predominantly through activity against the HCN4 channel (Ide et al., 2019). Importantly, ivabradine is a well-tolerated drug used in the treatment of heart failure and angina, with few major side effects reported (Koruth et al., 2017). Furthermore, Org 34,167 has a brain to blood plasma ratio of ∼17, potentially limiting peripheral side effects. However, compounds with limited inhibition of HCN4 channels would further reduce the impact on the heart.

Org 34,167 successfully completed phase 1 trials for depression and was reported to be well tolerated at doses as high as 40 mg (Slattery, 2003). Org 34,167 was also in early clinical development as an anaesthetic agent (AdisInsight, 2009). No recent information regarding the compound has been published, suggesting that development has been discontinued for both indications, and the reason why the two trials were discontinued is not publicly available. Side effects reported are relatively mild and include dizziness, impaired concentration, nausea and vomiting, sleeping disturbances, vertigo, fatigue, headache, palpitations and visual disturbances (Slattery, 2003). However, our preclinical data suggest a narrow therapeutic window in mice, with tremors emerging at doses only marginally higher than the effective dose. It is worth noting that tremors and a proconvulsant propensity were reported in rats at doses greater than 0.5 mg/kg (Slattery, 2003). As discussed, tremors are likely due to HCN2 channel inhibition and compounds with less activity against this channel would be predicted to have a larger therapeutic window.

A limitation of this study is that we have not explored potential off-target activity of Org 34,167. Whilst the data is not presented, Slattery states that Org 34,167 only displayed high affinity for HCN channels with weak affinity for sodium channels (Slattery, 2003). Furthermore, Org 34,167 is specifically mentioned in the patent application US20110092486A1 as an *I*_h_ channel inhibitor (Ward et al., 2011). Slattery also states that Org 34,167 displayed low affinity for >50 G-protein coupled receptors and lacked effect on traditional anti-depressive targets (Slattery, 2003). Nevertheless, it is possible that the anti-depressive activity may be occurring through a distinct mechanism, and this warrants further investigation.

While the lack of impact of Org 34,167 on C57BL/6J mice is unclear, as previously mentioned there is evidence that C57BL/6J mice do not respond appropriately to antidepressants such as fluoxetine when undergoing tests to assess depressive-like behaviour in comparison with BALB/c mice (Lucki et al., 2001; Jacobson and Cryan, 2007). Specifically, Lucki et al., 2001 reported that BALB/c mice display an appropriate anti-depressive response to the Porsolt swim test when administered fluoxetine (10 mg/kg), whereas C57BL/6J mice display no change in time spent immobile. The biological underpinning of these behavioural differences is unknown, though differences in serotonergic and/or dopaminergic neurochemistry have been suggested (Jacobson and Cryan, 2007). As such, the ability of Org 34,167 to reduce depressive-like behaviours across three tests with a similar efficacy to fluoxetine in both male and female BALB/c mice is consistent with its anti-depressive effects.

Although a wide variety of pharmacological treatments are used to clinically manage depression, one-half to two-thirds of patients may be resistant to routinely administered antidepressants (Fava, 2003; Rush et al., 2006; Warden et al., 2007), such as tricyclic antidepressants, monoamine oxidase inhibitors and SSRIs, and do not respond adequately to standard treatment regimes. As such, the exploration and development of novel antidepressant compounds with alternative mechanisms of action are essential for addressing the needs of these patients, especially when considering both the prevalence and the tremendous social, financial and health burdens of depression (Mrazek et al., 2014; Herrman et al., 2022).

In conclusion, we present data demonstrating that a small molecule, which inhibits HCN channels in a broad-spectrum manner, has antidepressant-like activity in mice. Physiological studies suggest that HCN1 channels are plausibly the primary basis of efficacy, while HCN2 and HCN4 channels associate with side effects, including tremor and bradycardia respectively, at higher doses. As such, compounds with greater HCN channel isoform selectivity are needed to further assess how HCN subtype activity impacts both efficacy and side effects, and these compounds may be more efficacious as antidepressant drugs with better tolerability profiles.

## Data Availability

The original contributions presented in the study are included in the article/Supplementary Material, further inquiries can be directed to the corresponding author.
