# Supplementary material for: Antidepressant-like activity of a brain penetrant HCN channel inhibitor in mice
Source: Front Pharmacol. 2023 May 10;14:1159527. doi: 10.3389/fphar.2023.1159527 (PMC10206048; doi:10.3389/fphar.2023.1159527)
Supplement: Supplementary file 2 [file Table1.docx]

***Supplementary Material***

**Antidepressant-like activity of a brain penetrant HCN channel inhibitor in mice**

Paulo Pinares-Garcia^†^, James Spyrou^†^, Chaseley E. McKenzie, Ian C. Forster, Ming S. Soh, Erlina Mohamed Syazwan, Mohammed Atif, Christopher A. Reid^*^

^†^These authors share first authorship.

*Corresponding Author: [Christopher.Reid@florey.edu.au](mailto:Christopher.Reid@florey.edu.au)

**Supplementary Figures and Tables**

**Supplementary Table 1.** Exposure parameters for Org 34167 in male C57BL/6J mice following intraperitoneal administration at 0.5 mg/kg. C_max_ = peak plasma concentration, T_max_ = time to C_max_, Apparent t_1/2_ = estimated half-life, AUC = area under the concentration-time curve.

| **C_max_ (µM)** | **T_max_ (h)** | **Apparent t_1/2_ (h)** | **AUC_0-last_ (h*µM)** | **AUC_0-inf_ (h*µM)** | **AUC_0-30min_ (h*µM)** |
| --- | --- | --- | --- | --- | --- |
| 0.103 | 0.25 | 0.78 | 0.0950 | 0.098 | 0.0369 |

**Supplementary Table 2.** Summary of one factor analyses of variance for locomotion, ledged beam, rotarod, marble burying, Porsolt swim, tail suspension, and heart rate tests. df = degrees of freedom; PST = Porsolt swim test; TST = tail suspension test. F-statistic is listed under the F / MS column in the Treatment row, and MS_residual_ is listed under the F / MS column in the Residual row.

[See supplementary file: Table 2.xls]


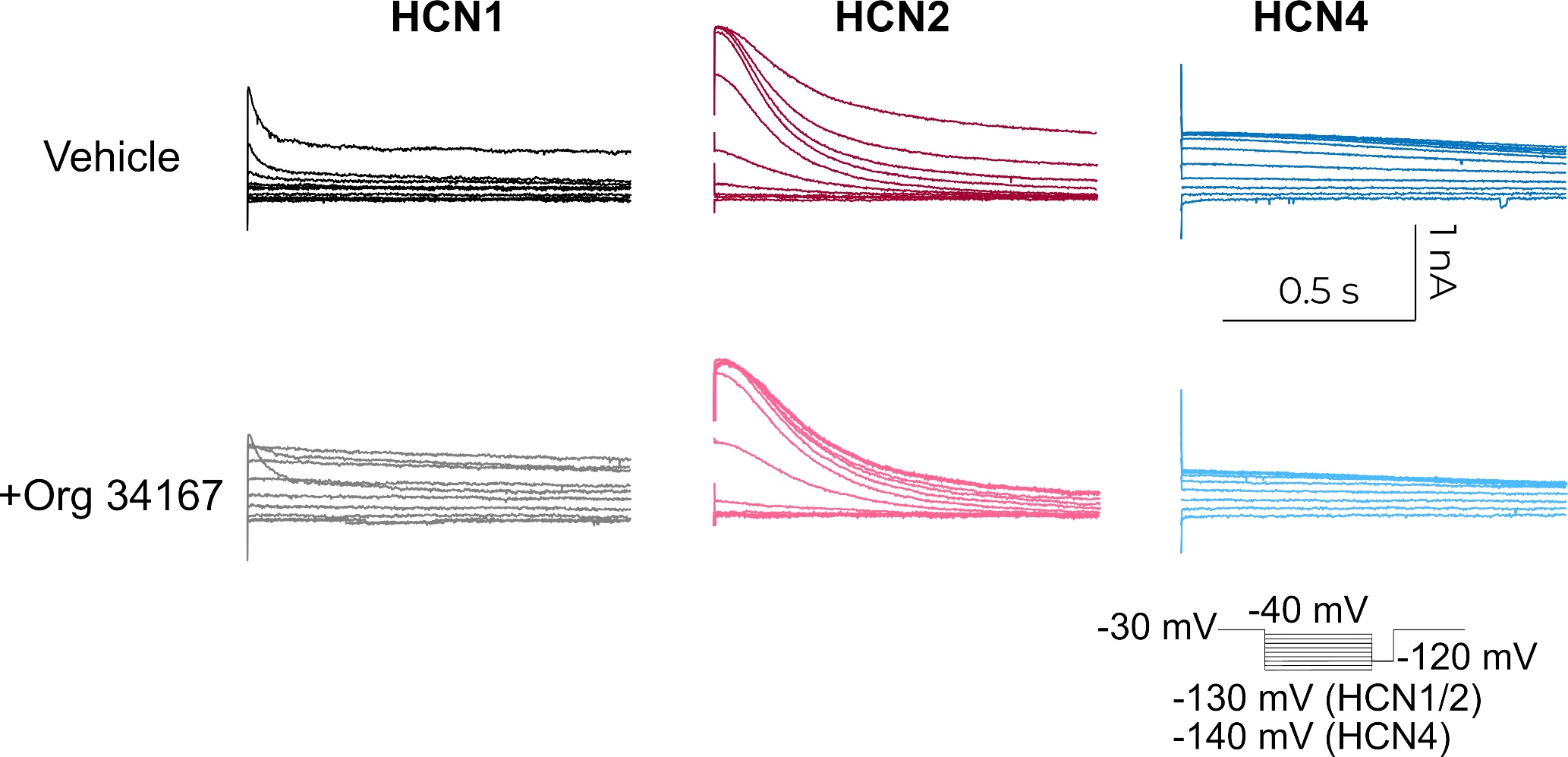


**Supplementary Figure 1.** Representative tail current traces for vehicle (top panel) and Org 34167 (bottom panel) on HCN1, HCN2 and HCN4 channels, elicited from the activation protocol shown below the HCN4 traces.


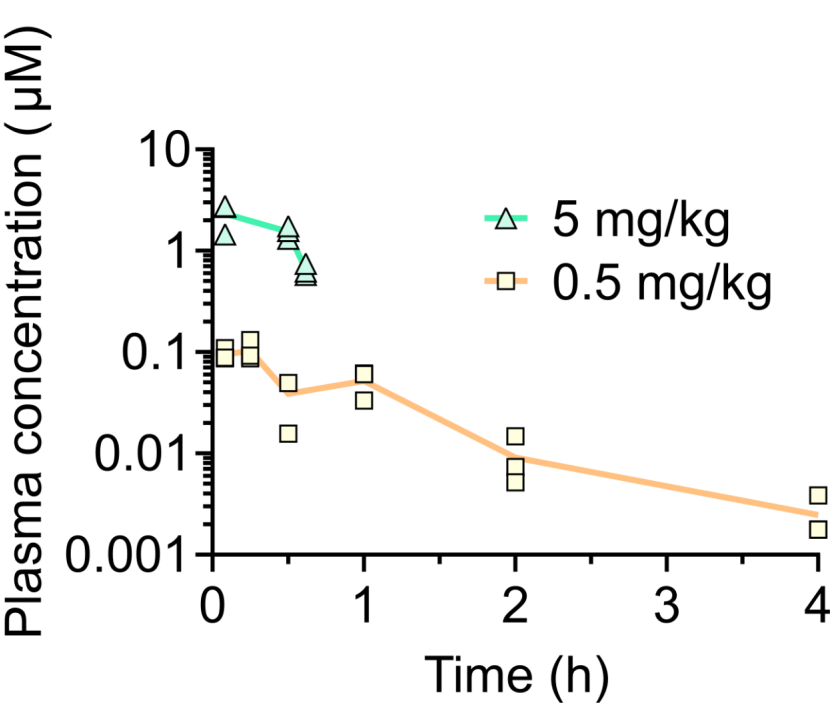


**Supplementary Figure 2.** Pharmacokinetic properties of Org 34167 in C57BL/6J mice. Plasma concentrations of Org 34167 in male C57BL/6J mice (21.5-25 g) following intraperitoneal administration at 0.5 and 5 mg/kg. Sampling for 5 mg/kg was terminated at 37 minutes post-dose due to the observation of adverse reactions.


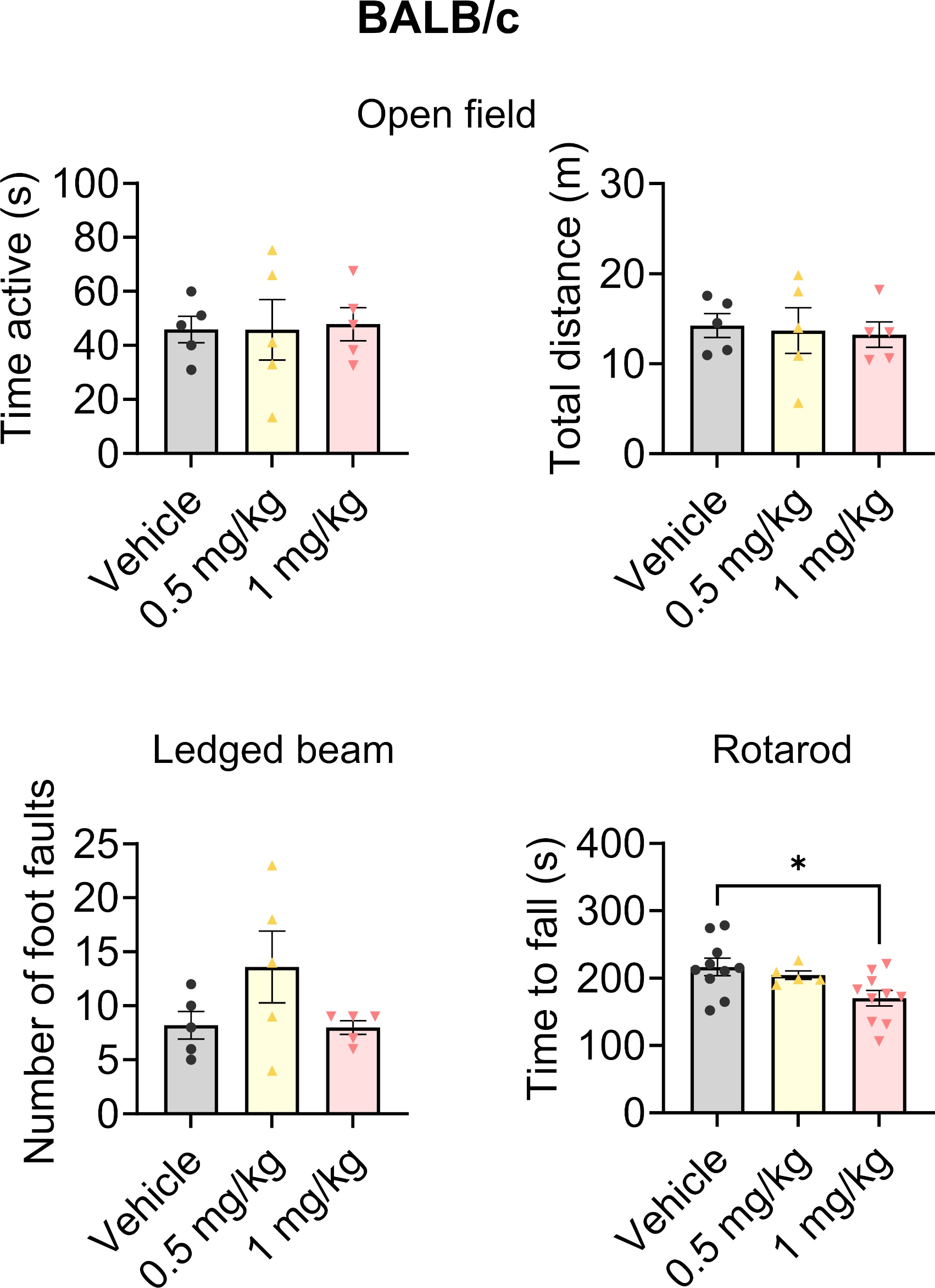


**Supplementary Figure 3.** Effect of Org 34167 on time active and total distance travelled in open field test (n = 5/group), number of foot faults in ledged beam test (n = 5/group), and time to fall in rotarod test (n = 5-10/group), in female BALB/c mice. *P < 0.05.
